# Subgroup identification of early preterm birth (ePTB): informing a future prospective enrichment clinical trial design

**DOI:** 10.1186/s12884-016-1189-0

**Published:** 2017-01-10

**Authors:** Chuanwu Zhang, Lili Garrard, John Keighley, Susan Carlson, Byron Gajewski

**Affiliations:** 1 Department of Biostatistics, University of Kansas Medical Center, Mail Stop 1026, 3901 Rainbow Blvd., Kansas City, KS 66160 USA; 2 Division of Biometrics III, OB/OTS/CDER, U.S. Food and Drug Administration, Silver Spring, MD 20993 USA; 3 Department of Dietetics and Nutrition, School of Health Professions, University of Kansas Medical Center, Mail Stop 1026, 3901 Rainbow Blvd., Kansas City, KS 66160 USA

**Keywords:** Early preterm birth, Risk factor, Interaction, Classification and regression tree, Logistic regression, Enrichment trial design

## Abstract

**Background:**

Despite the widely recognized association between the severity of early preterm birth (ePTB) and its related severe diseases, little is known about the potential risk factors of ePTB and the sub-population with high risk of ePTB. Moreover, motivated by a future confirmatory clinical trial to identify whether supplementing pregnant women with docosahexaenoic acid (DHA) has a different effect on the risk subgroup population or not in terms of ePTB prevalence, this study aims to identify potential risk subgroups and risk factors for ePTB, defined as babies born less than 34 weeks of gestation.

**Methods:**

The analysis data (*N =* 3,994,872) were obtained from CDC and NCHS’ 2014 Natality public data file. The sample was split into independent training and validation cohorts for model generation and model assessment, respectively. Logistic regression and CART models were used to examine potential ePTB risk predictors and their interactions, including mothers’ age, nativity, race, Hispanic origin, marital status, education, pre-pregnancy smoking status, pre-pregnancy BMI, pre-pregnancy diabetes status, pre-pregnancy hypertension status, previous preterm birth status, infertility treatment usage status, fertility enhancing drug usage status, and delivery payment source.

**Results:**

Both logistic regression models with either 14 or 10 ePTB risk factors produced the same C-index (0.646) based on the training cohort. The C-index of the logistic regression model based on 10 predictors was 0.645 for the validation cohort. Both C-indexes indicated a good discrimination and acceptable model fit. The CART model identified preterm birth history and race as the most important risk factors, and revealed that the subgroup with a preterm birth history and a race designation as Black had the highest risk for ePTB. The c-index and misclassification rate were 0.579 and 0.034 for the training cohort, and 0.578 and 0.034 for the validation cohort, respectively.

**Conclusions:**

This study revealed 14 maternal characteristic variables that reliably identified risk for ePTB through either logistic regression model and/or a CART model. Moreover, both models efficiently identify risk subgroups for further enrichment clinical trial design.

## Background

Preterm birth, also known as premature birth, is the birth of a baby at less than 37 weeks of gestational age (http://www.who.int/mediacentre/factsheets/fs363/en/, http://www.cdc.gov/reproductivehealth/maternalinfanthealth/pretermbirth.htm). Preterm birth occurs in 9.57% of all U.S. births each year [1]. Worldwide, approximately 15 million babies are born prematurely each year (http://www.who.int/mediacentre/factsheets/fs363/en/). Preterm birth increases the risk of many severe health outcomes. Infants born preterm are more likely to experience early death than are infants born at term [2, 3]; and preterm birth is the leading cause of both neonatal death and long-term neurological disabilities for children in the United States (http://www.cdc.gov/reproductivehealth/maternalinfanthealth/pretermbirth.htm) [4]. Moreover, adults who were born preterm are at increased risk of having hypertension [5, 6], mental health disorders, chronic respiratory disease, and neurologic and learning disabilities [7]. Preterm birth causes great social and medical burdens both in the U.S. [8, 9] and worldwide [10–12]. Early preterm birth (ePTB)—birth at less than 34 weeks—has the highest risk of mortality and other diseases in adulthood [13, 14]. The importance of prevention is evident for preterm birth, including ePTB. Consequently, to identify the risk factors of preterm birth, especially for ePTB, is a highly important step that will provide valuable information for subsequent enrichment clinical trial designs of targeted preventions and/or treatment.

Several recent studies have explored the risk factors for ePTB [15–18]. Researchers have identified a few potential maternal risk factors associated with preterm birth including maternal hypertension [5], Factor V Leiden [19], lower genital tract inflammatory milieu [20], prior preeclampsia [18], and Crohn’s disease [21]. Not only were these trials limited in statistical power, few studies explored potential risk factors for ePTB, which has a higher risk for poor health outcomes [13, 22]. In addition, interaction among the risk factors was typically not considered, despite the important role played by the interaction among risk factors in the prevention and treatment of preterm birth, including ePTB. From a practical perspective, this analysis is motivated by a desire to inform a future confirmatory clinical trial designed to identify whether supplementing pregnant women with docosahexaenoic acid (DHA) can differently reduce the rate of ePTB for the subgroups. DHA supplementation provides a high yield, low risk provocative strategy to reduce ePTB delivery in the U.S. by up to 75% [23]. However, little is known regarding the effect profile of DHA on various populations; and it is possible for DHA to have different effects on different risk subgroups.

Based on findings from previous studies on preterm birth and our future research interest, the specific aim for this study is to identify potential risk subgroups and risk factors for the main outcome, ePTB, defined previously as babies born prior to 34 weeks of gestation [14, 24]. We applied and compared both logistic regression and classification and regression tree (CART) models to identify potential risk subgroups and risk factors from maternal demographic characteristics [4, 25] and maternal pre-pregnancy characteristics for ePTB. To the author’s best knowledge, this is the first study to explore the association of ePTB with risk factors, the interactions among the risk factors, and to identify potential subgroups to inform future enrichment trial designs.

## Methods

### 2014 natality public data file

The ePTB population data used for these analyses were obtained from the National Vital Statistics System’s 2014 Natality public data file, compiled by the Centers for Disease Control and Prevention’s (CDC) National Center for Health Statistics (NCHS). Since federal law mandates national collection and publication of births and other vital statistical data, all births occurring and registered within the U.S. in 2014 were collected directly from the 50 U.S. states, New York City, and the District of Columbia (DC) [26]. The overall database contains 3,998,175 records comprised of demographic characteristics of the mother, father, and the child (e.g., gestation), maternal prenatal care, pregnancy history, and health data, etc. The public data and the corresponding user’s guide are available from the website: http://www.cdc.gov/nchs/data_access/Vitalstatsonline.htm

### Study population

After excluding 3303 cases for which the gestation period from the original 2014 Natality public data file was unknown, the final analysis file for the current study included 3,994,872 records. Since the main outcome variable is ePTB, a binary flag variable representing the ePTB status (i.e., 1 = < 34 weeks: ePTB and 0 = ≥ 34 weeks) was created in the analysis file. The analysis file included selected maternal demographic characteristics considered relevant to ePTB, such as mothers’ age, mothers’ nativity, mothers’ race, mothers’ Hispanic origin, marital status, mothers’ education, delivery payment source. Delivery payment source was included as an additional covariate that may provide additional information on the implications of socioeconomic status for ePTB. Maternal pre-pregnancy characteristics and medical history were also included in the ePTB risk factor analysis. These factors included smoking status, body mass index (BMI), diabetes status, hypertension status, previous preterm birth status, infertility treatment usage status and fertility enhancing drug usage status. In total, 14 maternal variables from the database were used as risk predictors in statistical models. The father’s demographic characteristics were not considered for this study.

A total of 142,851 (3.58%) observations from the analysis file contained at least one missing value for some of the predictors and those predictors were categorized as “missing.” Predictors with responses of “Unknown,” “Not Stated,” “Not Applicable,” and “Other,” were categorized together as shown in the descriptive statistics listed in Tables 1 and 2.

**Table 1:** Subject demography information

Variable	Newborn Gestational Age	
	< 34 weeks: ePTB	≥ 34 weeks
	*N* = 134009	*N* = 3860863
Mothers’ Age (%)		
≤ 24 Years	40711 (30.38)	1094793 (28.36)
25-29 Years	34831 (25.99)	1112643 (28.82)
30-34 Years	33578 (25.06)	1049775 (27.19)
≥ 35 Years	24889 (18.57)	603652 (15.64)
Mothers’ Nativity (%)		
Born in U.S.	107578 (80.28)	2996531 (77.61)
Born Outside U.S. /Unknown/Not Stated	26431 (19.72)	864332 (22.39)
Mothers’ Race (%)		
White	88185 (65.81)	2938466 (76.11)
Black	36554 (27.28)	603921 (15.64)
American Indian/Alaskan Native/Asian or Pacific Islander	9270 (6.92)	318476 (8.25)
Mothers’ Hispanic Origin (%)		
Non-Hispanic/Hispanic Origin Not Stated	105011 (78.36)	2968422 (76.88)
Hispanic	28998 (21.64)	892441 (23.12)
Marital Status (%)		
Married	65594 (48.95)	2323620 (60.18)
Unmarried	68415 (51.05)	1537243 (39.82)
Mothers’ Education (%)		
≤ High School or GED/Unknown	62819 (46.88)	1512489 (39.17)
Associate/Some College Credit	37338 (27.86)	1086153 (28.13)
≥ Bachelor's	29145 (21.75)	1124077 (29.11)
Missing	4707 (3.51)	138144 (3.58)
Pre-pregnancy Smoking Status (%)		
Nonsmoker	108663 (81.09)	3258557 (84.40)
Smoker/Unknown/Not Stated	20639 (15.40)	464162 (12.02)
Missing	4707 (3.51)	138144 (3.58)
Pre-pregnancy BMI (%)		
Under Weight-Normal ≤ 24.9	55824 (41.66)	1785913 (46.26)
Overweight 25.0-29.9	30288 (22.60)	918380 (23.79)
Obesity ≥ 30.0/Unknown/Not Stated	43190 (32.23)	1018426 (26.38)
Missing	4707 (3.51)	138144 (3.58)
Pre-pregnancy Diabetes Status (%)		
No/Unknown/Not Stated	126901 (94.70)	3694967 (95.70)
Yes	2401 (1.79)	27752 (0.72)
Missing	4707 (3.51)	138144 (3.58)
Pre-pregnancy Hypertension Status (%)		
No/Unknown/Not Stated	123932 (92.48)	3667289 (94.99)
Yes	5370 (4.01)	55430 (1.44)
Missing	4707 (3.51)	138144 (3.58)
Previous Preterm Birth Status (%)		
No/Unknown/Not Stated	118468 (88.40)	3626879 (93.94)
Yes	10834 (8.08)	95840 (2.48)
Missing	4707 (3.51)	138144 (3.58)
Infertility Treatment Usage Status (%)		
No/Unknown/Not Stated	122859 (91.68)	3669850 (95.05)
Yes	6443 (4.81)	52869 (1.37)
Missing	4707 (3.51)	138144 (3.58)
Fertility Enhancing Drug Usage Status (%)		
No/Not Applicable/Unknown/Not Stated	126582 (94.46)	3697856 (95.78)
Yes	2720 (2.03)	24863 (0.64)
Missing	4707 (3.51)	138144 (3.58)
Delivery Payment Source (%)		
Medicaid	65048 (48.54)	1598851 (41.41)
Private Insurance	51753 (38.62)	1771814 (45.89)
Self-pay/Other/Unknown	12501 (9.33)	352054 (9.12)
Missing	4707 (3.51)	138144 (3.58)

**Table 2:** Univariate difference between training sample and validation sample

Variables	Cohort	
	Training	Validation
	*N* = 2796411	*N* = 1198461
Mothers’ Age (%)		
≤ 24 Years	794486 (28.41)	341018 (28.45)
25-29 Years	803113 (28.72)	344361 (28.73)
30-34 Years	758087 (27.11)	325266 (27.14)
≥ 35 Years	440725 (15.76)	187816 (15.67)
Mothers’ Nativity (%)		
Born in U.S.	2172903 (77.70)	931206 (77.70)
Born Outside U.S. /Unknown/Not Stated	623508 (22.30)	267255 (22.30)
Mothers’ Race (%)		
White	2119115 (75.78)	907536 (75.73)
Black	447972 (16.02)	192503 (16.06)
American Indian/Alaskan Native/Asian or Pacific Islander	229324 (8.20)	98422 (8.21)
Mothers’ Hispanic Origin (%)		
Non-Hispanic/Hispanic Origin Not Stated	2151766 (76.95)	921667 (76.90)
Hispanic	644645 (23.05)	276794 (23.10)
Marital Status (%)		
Married	1672583 (59.81)	716631 (59.80)
Unmarried	1123828 (40.19)	481830 (40.20)
Mothers’ Education (%)		
≤ High School or GED/Unknown	1102757 (39.43)	472551 (39.43)
Associate/Some College Credit	786618 (28.13)	336873 (28.11)
≥ Bachelor's	806822 (28.85)	346400 (28.90)
Missing	100214 (3.58)	42637 (3.56)
Pre-pregnancy Smoking Status (%)		
Nonsmoker	2357285 (84.30)	1009935 (84.27)
Smoker/Unknown/Not Stated	338912 (12.12)	145889 (12.17)
Missing	100214 (3.58)	42637 (3.56)
Pre-pregnancy BMI (%)		
Under Weight-Normal ≤ 24.9	1288811 (46.09)	552926 (46.14)
Overweight 25.0-29.9	664673 (23.77)	283995 (23.70)
Obesity ≥ 30.0/Unknown/Not Stated	742713 (26.56)	318903 (26.61)
Missing	100214 (3.58)	42637 (3.56)
Pre-pregnancy Diabetes Status (%)		
No/Unknown/Not Stated	2675048 (95.66)	1146820 (95.69)
Yes	21149 (0.76)	9004 (0.75)
Missing	100214 (3.58)	42637 (3.56)
Pre-pregnancy Hypertension Status (%)		
No/Unknown/Not Stated	2653410 (94.89)	1137811 (94.94)
Yes	42787 (1.53)	18013 (1.50)
Missing	100214 (3.58)	42637 (3.56)
Previous Preterm Birth Status (%)		
No/Unknown/Not Stated	2621496 (93.75)	1123851 (93.77)
Yes	74701 (2.67)	31973 (2.67)
Missing	100214 (3.58)	42637 (3.56)
Infertility Treatment Usage Status (%)		
No/Unknown/Not Stated	2654757 (94.93)	1137952 (94.95)
Yes	41440 (1.48)	17872 (1.49)
Missing	100214 (3.58)	42637 (3.56)
Fertility Enhancing Drug Usage Status (%)		
No/Not Applicable/Unknown/Not Stated	2676910 (95.73)	1147528 (95.75)
Yes	19287 (0.69)	8296 (0.69)
Missing	100214 (3.58)	42637 (3.56)
Delivery Payment Source (%)		
Medicaid	1164617 (41.65)	499282 (41.66)
Private Insurance	1276362 (45.64)	547205 (45.66)
Self-pay/Other/Unknown	255218 (9.13)	109337 (9.12)
Missing	100214 (3.58)	42637 (3.56)
Newborn Gestational Age (%)		
< 34 weeks: ePTB	93751 (3.35)	40258 (3.36)
≥ 34 weeks	2702660 (96.65)	1158203 (96.64)

### Statistical analysis

#### Training and validation datasets

The large sample size allowed for independent training and validation cohorts. The overall sample was divided randomly into a training cohort (70%) and a validation cohort (30%), stratifying by ePTB status to ensure a balanced partition. Descriptive statistics were summarized to compare the demographic and pre-pregnancy information between the two cohorts of data. The training sample was used to build models via both logistic regression and CART and the validation sample was used to evaluate the models obtained from the training cohort.

#### Logistic regression

In order to investigate the association of ePTB with the potential risk factors, a multivariate logistic regression model was applied to estimate odds ratios (OR) and the corresponding 95% confidence intervals (CI). All predictors entered the model and they were selected via backward elimination. We set the significance level to stay in the model for a predictor to 0.05. A further simplified logistic regression model was fitted using 10 covariates to explore risk subgroups of ePTB. The predicted probabilities were calculated for the validation cohort based on the simplified model obtained from the training cohort. Based on the validation cohort, the calibration plot was generated to compare the average predicted probabilities and the average observed probabilities. The c-index was calculated to identify the model discriminatory capacity in terms of the training and validation cohorts.

#### CART model

CART model can be a very useful complement to a logistic regression model because the CART model can identify unknown interactions among the risk factors of ePTB. CART is a nonparametric method that derives hidden patterns in data by constructing a series of binary splits on the outcome of interest [27–29]. The most discriminating predictor is selected to form the first partition based on the ability of the variables to minimize the within-group variance of the dependent variable, so the observations within each subgroup share the same characteristics that influence the probability of belonging to the interested response group [30]. This step is executed repeatedly to each partition until the sample size of each subgroup (i.e., a terminal node) is at or below a pre-specified level. In this study, the terminal node was specified as 0.5% of the total sample (either the training sample or the validation sample). A maximum tree first was constructed and standard pruning strategies were then applied to arrive at a parsimonious tree with a low misclassification rate and a high discriminatory capacity [31]. The final CART model can be visualized as an upside-down tree with the parent node of the tree containing the entire sample. Additional child nodes can be created using the Gini splitting rule for binary outcomes [32], and the terminal nodes are where predictions and inferences are made. The training cohort was used to generate an appropriate CART tree, and the validation cohort was utilized to evaluate the CART tree via the C-index and the misclassification rate.

All statistical tests were two-tailed with *p* ≤ 0.05 as the statistically significant level. The CART analysis was executed in SAS Enterprise Miner Workstation 13.1 [32], and all other statistical analyses and the data management were conducted with SAS 9.4.

## Results

### Characteristics of the study population and training and validation datasets

As previously mentioned, the analysis file included 3,994,872 records which contained 134,009 cases of ePTB (<34 weeks) and 3,860,863 cases of baby birth ≥ 34 weeks of gestation. The characteristics of the subjects stratified by ePTB status are shown in Table 1. For the training and validation cohorts, 70% (*N* = 2,796,411) and 30% (*N* = 1,198,461) of the total sample were generated for each cohort, respectively. The frequencies and related percentages of each predictor were similar after the random split stratified by the ePTB status, indicating that the partition is well-balanced (Table 2).

### Logistic regression

#### 14-predictor model

Table 3 showed results from the logistic regression analysis for prevalence of ePTB with all 14 predictor variables. A relatively higher ePTB prevalence was observed in the older mother populations compared to younger mothers in the ≤ 24 years old reference group. The adjusted OR (95% CI) were 1.013 (0.995, 1.032), 1.130 (1.108, 1.152), and 1.354 (1.325, 1.385) for mothers in the age groups of 25-29 years (non-significant, *p* = 0.169), 30-34 years, and ≥ 35 years, respectively. Mothers born outside of the U.S. were less likely to experience ePTB compared to mothers born in the U.S. with an adjusted OR (95% CI) of 0.880 (0.863, 0.898). Black mothers and American Indian/Alaskan Native/Asian or Pacific Islander mothers were more likely to have an ePTB compared to White mothers with adjusted OR (95% CI) of 1.773 (1.743, 1.803) and 1.096 (1.066, 1.127), respectively. Mothers of Hispanic origin had a slightly higher ePTB prevalence compared to mothers of non-Hispanic origin with an adjusted OR (95% CI) of 1.033 (1.013, 1.053). ePTB was more likely to occur in the unmarried mother population compared to married mothers with an adjusted OR (95% CI) of 1.326 (1.304, 1.347).

**Table 3:** The estimate and adjusted OR of logistic regression analysis on the training cohort

Parameter	Estimate	Adjusted OR (95% CI)	*P* value
Intercept	-3.7154	-	<.0001
Mothers’ Age (%)			
≤ 24 Years	-	1.0 (1.0–1.0)	-
25-29 Years	0.0129	1.013 (0.995, 1.032)	0.169
30-34 Years	0.1221	1.130 (1.108, 1.152)	<.0001
≥ 35 Years	0.3034	1.354 (1.325, 1.385)	<.0001
Mothers’ Nativity (%)			
Born in U.S.	-	1.0 (1.0–1.0)	-
Born Outside U.S. /Unknown/Not Stated	-0.1274	0.880 (0.863, 0.898)	<.0001
Mothers’ Race (%)			
White	-	1.0 (1.0–1.0)	-
Black	0.5727	1.773 (1.743, 1.803)	<.0001
American Indian/Alaskan Native/Asian or Pacific Islander	0.0917	1.096 (1.066, 1.127)	<.0001
Mothers’ Hispanic Origin (%)			
Non-Hispanic/Hispanic Origin Not Stated	-	1.0 (1.0–1.0)	-
Hispanic	0.0323	1.033 (1.013, 1.053)	0.009
Marital Status (%)			
Married	-	1.0 (1.0–1.0)	-
Unmarried	0.2819	1.326 (1.304, 1.347)	<.0001
Mothers’ Education (%)			
≤ High School or GED/Unknown	-	1.0 (1.0–1.0)	-
Associate/Some College Credit	-0.1725	0.842 (0.828, 0.856)	<.0001
≥ Bachelor's	-0.3382	0.713 (0.698, 0.729)	<.0001
Missing	0.0031	1.003 (0.966, 1.042)	0.8727
Pre-pregnancy Smoking Status (%) ^a^			
Nonsmoker	-	1.0 (1.0–1.0)	-
Smoker/Unknown/Not Stated	0.1677	1.183 (1.160, 1.206)	<.0001
Pre-pregnancy BMI (%) ^a^			
Under Weight-Normal ≤24.9	-	1.0 (1.0–1.0)	-
Overweight 25.0-29.9	-0.0174	0.983 (0.966, 1.000)	0.0472
Obesity ≥30.0/Unknown/Not Stated	0.1195	1.127 (1.109, 1.145)	<.0001
Pre-pregnancy Diabetes Status (%) ^a^			
No/Unknown/Not Stated	-	1.0 (1.0–1.0)	-
Yes	0.5741	1.776 (1.685, 1.871)	<.0001
Pre-pregnancy Hypertension Status (%) ^a^			
No/Unknown/Not Stated	-	1.0 (1.0–1.0)	
Yes	0.6849	1.984 (1.913, 2.056)	<.0001
Previous Preterm Birth Status (%) ^a^			
No/Unknown/Not Stated	-	1.0 (1.0–1.0)	-
Yes	1.0999	3.004 (2.929, 3.081)	<.0001
Infertility Treatment Usage Status (%) ^a^			
No/Unknown/Not Stated	-	1.0 (1.0–1.0)	-
Yes	1.6299	5.103 (4.888, 5.328)	<.0001
Fertility Enhancing Drug Usage Status (%) ^a^			
No/Not Applicable/Unknown/Not Stated	-	1.0 (1.0–1.0)	-
Yes	-0.1988	0.820 (0.769, 0.873)	<.0001
Delivery Payment Source (%) ^a^			
Medicaid	-	1.0 (1.0–1.0)	-
Private Insurance	-0.0352	0.965 (0.948, 0.983)	<.0001
Self-pay/Other/Unknown	0.0762	1.079 (1.054, 1.105)	<.0001

^a^: For the following parameters after mothers’ education, missing observations were automatically excluded from the analysis, and the corresponding parameters were automatically set to 0 due to they are from the same subset

Mothers with an associate degree or some college credit and mothers with a bachelor’s degree or higher education were less likely to experience ePTB compared to mothers with a high school/general educational development (GED) or less education. The corresponding adjusted OR (95% CI) for each subgroup was 0.842 (0.828, 0.856) and 0.713 (0.698, 0.729), respectively. Results from the subgroup with missing mother’s education were non-significant (*p* = 0.873). In addition, since all the observations with missing predictors were all from the same subset, for the following parameters after mothers’ education, missing observations were automatically excluded from the analysis, and the corresponding parameters were automatically set to 0 due to they are from the same subset.

Some maternal pre-pregnancy characteristics and medical history factors were also found to be related to ePTB. For Pre-pregnancy BMI, mothers in the overweight subgroup had a slightly lower prevalence of ePTB (*p* = 0.047), with an adjusted OR (95% CI) of 0.983 (0.966, 1.000) compared to mothers with underweight and/or normal BMI. However, the opposite result was obtained for the obese subgroup with an adjusted OR (95% CI) of 1.127 (1.109, 1.145), compared with the underweight and/or normal BMI mothers. For other pre-pregnancy risk factors (i.e., smoking status, diabetes status, hypertension status, and previous preterm birth status), mothers in each risk sub-category were more likely to have a higher prevalence of ePTB compared to mothers who did not have the abovementioned risk factors. The corresponding adjusted OR (95% CI) were 1.183 (1.160, 1.206), 1.776 (1.685, 1.871), 1.984 (1.913, 2.056), 3.004 (2.929, 3.081), respectively.

In addition, mothers who used infertility treatment were much more likely to experience ePTB than those who had not used the infertility treatment, with an adjusted OR (95% CI) of 5.103 (4.888, 5.328). On the other hand, a different outcome was observed with the usage of fertility enhancing drug. Mothers who used fertility enhancing drugs were less likely to have an ePTB compared to women who did not, with an adjusted OR (95% CI) of 0.820 (0.769, 0.873). Compared to women whose payer was Medicaid, the adjusted OR (95% CI) were 0.965 (0.948, 0.983) and 1.079 (1.054, 1.105) for women who had private insurance and self-pay, respectively. Mothers with private insurance had a slightly lower prevalence of ePTB; whereas mothers with self-paid delivery had a slightly higher prevalence of ePTB. Although the *p-*values for both comparisons were statistically significant (<0.0001), the numerical differences were small.

#### 10-predictor model

**Fig. 1:**
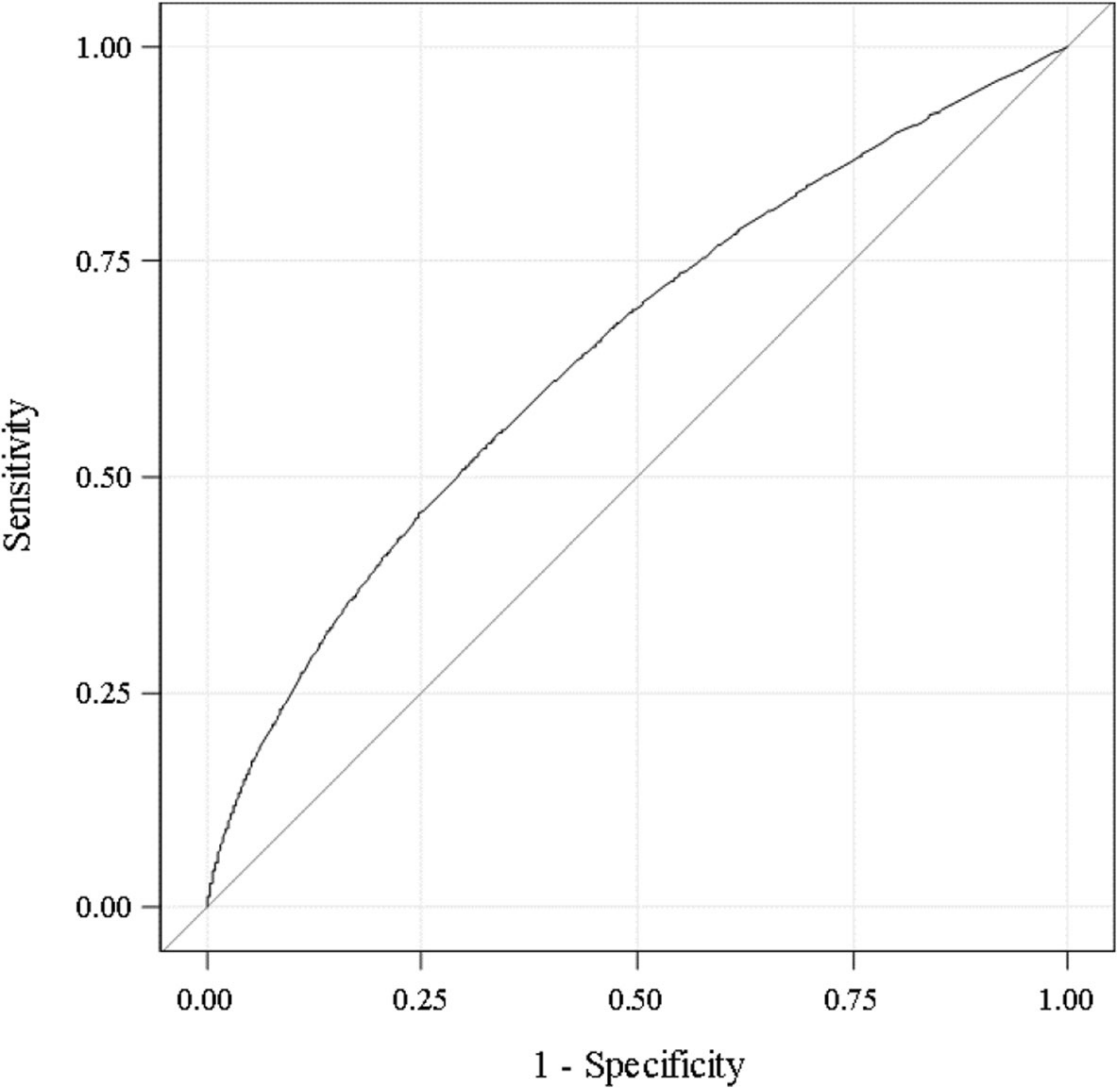
ROC curve from logistic regression on the training dataset (Area under the curve = 0.646)

**Fig. 2:**
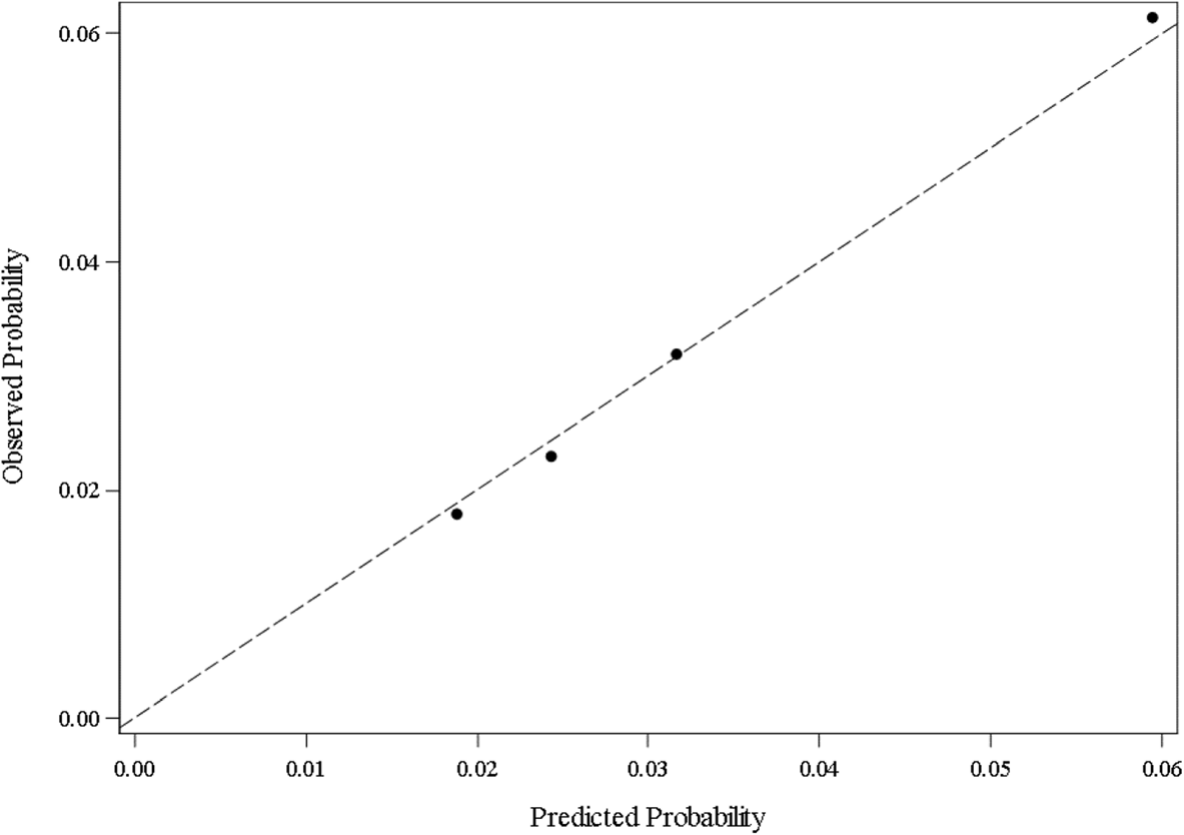
Calibration plot from the validation sample. Observed vs. Predicted Probability across the quartiles

After examining results from the 14-predictor model, four covariates - mothers’ nativity, mothers’ Hispanic origin, fertility enhancing drug usage status, and delivery payment source - were excluded for having minimal effects on ePTB and to explore further a smaller set of potential risk subgroups for ePTB. Moreover, the same C-index (0.646) was obtained from both logistic regression models with either 14 or 10 predictors based on the training cohort (Fig. 1). The C-index was 0.645 after fitting the 10-predictor model on the validation data, indicating an acceptable model fit. Figure 2 showed the calibration plot based on the validation cohort to compare the average predicted probabilities and the average observed probabilities across quartiles. The average and range of both predicted and observed probability for each of the four potential subgroups were shown in Table 4, along with summarized maternal characteristics for each subgroup from the validation cohort.

**Table 4:** The ePTB subgroup predicted /observed probability and maternal characteristics in validation cohort via logistic regression

Variable	Subgroup			
	1st Quartile	2nd Quartile	3rd Quartile	4th Quartile
	*N* = 299529	*N* = 299078	*N* = 299993	*N* = 299861
Probability (%)				
Average Predicted	1.92	2.46	3.22	6.02
Range Predicted	0.55	0.52	0.95	60.6
Average Observed	1.83	2.33	3.24	6.07
Mothers’ Age (%)				
≤ 24 Years	36603 (12.22)	70681 (23.63)	127739 (42.58)	105995 (35.35)
25-29 Years	120779 (40.32)	83600 (27.95)	68003 (22.67)	71979 (24.00)
30-34 Years	129538 (43.25)	78439 (26.23)	56362 (18.79)	60927 (20.32)
≥ 35 Years	12609 (4.21)	66358 (22.19)	47889 (15.96)	60960 (20.33)
Mothers’ Race (%)				
White	259978 (86.80)	273311 (91.38)	260128 (86.71)	114119 (38.06)
Black	0 (0.00)	872 (0.29)	18661 (6.22)	172970 (57.68)
American Indian/Alaskan Native/Asian or Pacific Islander	39551 (13.20)	24895 (8.32)	21204 (7.07)	12772 (4.26)
Marital Status (%)				
Married	296804 (99.09)	246717 (82.49)	92320 (30.77)	80790 (26.94)
Unmarried	2725 (0.91)	52361 (17.51)	207673 (69.23)	219071 (73.06)
Mothers’ Education (%)				
≤ High School or GED/Unknown	10988 (3.67)	93778 (31.36)	192086 (64.03)	175699 (58.59)
Associate/Some College Credit	69843 (23.32)	117843 (39.40)	69455 (23.15)	79732 (26.59)
≥ Bachelor’s	217614 (72.65)	71541 (23.92)	21886 (7.30)	35359 (11.79)
Missing	1084 (0.36)	15916 (5.32)	16566 (5.52)	9071 (3.03)
Pre-pregnancy Smoking Status (%)				
Nonsmoker	295313 (98.59)	262159 (87.66)	234907 (78.30)	217556 (72.55)
Smoker/Unknown/Not Stated	3132 (1.05)	21003 (7.02)	48520 (16.17)	73234 (24.42)
Missing	1084 (0.36)	15916 (5.32)	16566 (5.52)	9071 (3.03)
Pre-pregnancy BMI (%)				
Under Weight-Normal ≤ 24.9	183032 (61.11)	142007 (47.48)	119757 (39.92)	108130 (36.06)
Overweight 25.0-29.9	82956 (27.70)	67818 (22.68)	70451 (23.48)	62770 (20.93)
Obesity ≥ 30.0/Unknown/Not Stated	32457 (10.84)	73337 (24.52)	93219 (31.07)	119890 (39.98)
Missing	1084 (0.36)	15916 (5.32)	16566 (5.52)	9071 (3.03)
Pre-pregnancy Diabetes Status (%)				
No/Unknown/Not Stated	298445 (99.64)	283149 (94.67)	282480 (94.16)	282746 (94.29)
Yes	0 (0.00)	13 (0.00)	947 (0.32)	8044 (2.68)
Missing	1084 (0.36)	15916 (5.32)	16566 (5.52)	9071 (3.03)
Pre-pregnancy Hypertension Status (%)				
No/Unknown/Not Stated	298445 (99.64)	283162 (94.68)	282293 (94.10)	273911 (91.35)
Yes	0 (0.00)	0 (0.00)	1134 (0.38)	16879 (5.63)
Missing	1084 (0.36)	15916 (5.32)	16566 (5.52)	9071 (3.03)
Previous Preterm Birth Status (%)				
No/Unknown/Not Stated	298445 (99.64)	283162 (94.68)	283427 (94.48)	258817 (86.31)
Yes	0 (0.00)	0 (0.00)	0 (0.00)	31973 (10.66)
Missing	1084 (0.36)	15916 (5.32)	16566 (5.52)	9071 (3.03)
Infertility Treatment Usage Status (%)				
No/Unknown/Not Stated	298445 (99.64)	283162 (94.68)	283427 (94.48)	272918 (91.01)
Yes	0 (0.00)	0 (0.00)	0 (0.00)	17872 (5.96)
Missing	1084 (0.36)	15916 (5.32)	16566 (5.52)	9071 (3.03)

For the first subgroup (i.e., first quartile), the average predicted and observed probabilities were 1.92% and 1.83% respectively, with a range of 0.55% for the predicted probability. A typical mother from this potential subgroup was between 30-34 years old, with a designation as white, married, with a bachelor’s degree or higher education level, non-smoking, underweight to normal weight (BMI ≤24.9) before pregnancy, without notable pre-pregnancy risk factors (i.e., diabetes, hypertension, previous preterm birth), and without infertility treatment. The second subgroup (i.e., second quartile) had an average predicted and an average observed probability of 2.46% and 2.33% respectively, with a range of 0.52% for the predicted probability. Mothers from the second potential subgroup shared very similar characteristics with a typical mother from the first subgroup, with the exception of age (slightly younger, 25-29 years old) and slightly lower education level (associate degree or some college credit). The average and range of predicted probability for the third subgroup (i.e., third quartile) were 3.22% and 0.95%; and the observed probability was 3.24%. Similar to trends observed from the second subgroup (in comparison with the first subgroup), a typical mother from the third subgroup was younger (≤ 24 years old) and with less education (≤ high school or GED/unknown). Lastly, the average predicted and observed probabilities for the highest risk subgroup (i.e., last 25% of data) were 6.02% and 6.07% respectively, with the predicted probability range of 60.6%. Mothers in this high-risk subgroup exhibit much different characteristics from the other three subgroups. They tended to be younger (≤ 24 years old), Black, unmarried, with a high school/GED or less education level, and generally obese (≥ 30.0 BMI). Moreover, compared to the other three subgroups, a relatively higher percentage of mothers in this high-risk subgroup had pre-pregnancy diabetes, hypertension, previous preterm birth, and infertility treatment usage.

### CART model

**Fig. 3:**
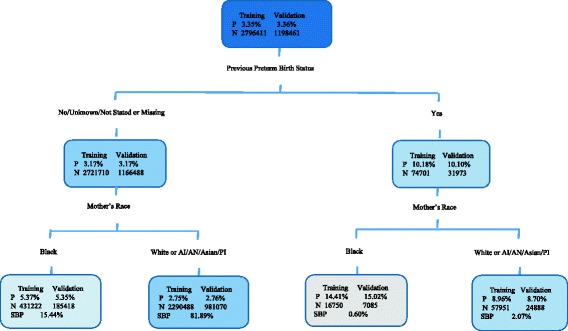
Classification and Regression Tree model for predicting ePTB. Legend: The probability of ePTB (P) and the number of subject (N) are all given inside of each node for both training and validation cohort. In each end node, the subgroup birth prevalence (SBP) is also calculated. AI = American Indian; AN = Alaskan Native; PI = Pacific Islander

For the CART model, sub-categories were collapsed for a couple of risk factors. The missing subgroup of previous preterm birth status was combined with the “no” group; and the race category of American Indian/Alaskan Native/Asian or Pacific Islander was combined with the White group. Based on a pre-specified stopping rule of having the terminal node size no less than 0.5% of the total sample and the binary Gini splitting rule, the CART tree was created to explore the unknown interactions among the risk factors and identify potential risk subgroups (Fig. 3). Overall, the CART model from the training cohort produced a misclassification rate of 0.034 and a C-index of 0.579. Moreover, the misclassification rate was 0.034 and the c-index was 0.578 from the validation cohort. By the percentage representing the observed prevalence of ePTB, CART identified four subgroups. Previous preterm birth status was identified as the most discriminating predictor for ePTB, followed by mothers’ race.

From training cohort, 14.41% of mothers with a preterm birth history and a race designation as Black had an ePTB experience (*n* =16,750), indicating a higher risk of ePTB for Black mothers with a preterm birth history. The correspondent percentage of this subgroup from the validation cohort is 15.02% (*n* = 7,085). This subgroup totally accounted for 0.60% of the overall 2014 U.S. births. 8.96% and 8.70% of mothers with a preterm birth history and a race designation as White had an ePTB experience from training (*n* = 57,951) and validation (*n* = 24,888), and the subgroup birth prevalence (SBP) was 2.07%. Women without a preterm birth history who were Black had an ePTB experience of 5.37% (*n* = 431,222); while 2.75% of mothers without a preterm birth history who were White had an ePTB experience (*n* = 2,290,488). The correspondent rates for the identical subgroups from the validation cohort are 5.35% (*n* =185,418) and 2.76% (*n* = 981,070). These two subgroups accounted for 15.44% and 81.89% of the overall birth data, respectively.

It is also informative to interpret the CART tree in terms of risk factors that increase or decrease the probability of ePTB. One can compare the rates of ePTB among the four potential subgroups to the average rate of ePTB of the total sample (3.35%, 3.36% for training and validation cohort, respectively). Three subgroups (with preterm birth history and Black, with preterm birth history and White, without preterm birth history and Black) had an increased probability of ePTB compared to the subgroup without a preterm birth history who were White.

## Discussion

This large sampled pioneer study aimed to explore potential risk factors and their interactions, and identify subgroup for the ePTB population via both logistic regression model and the CART model. Several important findings emerged from the current study. First, a subset of the most important and relevant covariates have been identified among the 14 risk factors examined, such as race, diabetes history, hypertension history, preterm birth history, and infertility treatment usage. Second, although logistic regression model identified a set of 10 predictors for the prevalence of ePTB, the CART model was able to examine multiple and complicated interactions among the selected predictors. The CART model clearly identified that the subgroup with a preterm birth history and a race designation as Black had the highest risk for ePTB. Third, although not presented in the current work, the risk ratios (RR) of a particular subgroup from the CART terminal nodes can be calculated to compare with the RR of other subgroups via the observed probabilities. RR also indirectly can inform the risk factors for ePTB.

Previous preterm birth status and race were the most discriminating predictors for ePTB by the CART model, while another eight predictors were identified by the logistic regression analyses. As a well-known traditional statistical approach, logistic regression provided predicted probabilities based on the important demographics and characteristics for ePTB; however, it cannot identify complicated interactions among risk factors. On the other hand, the CART model presents a more straightforward picture of the potential high risk subgroups for ePTB for whom targeted prevention efforts can be implemented. Moreover, each subgroup accounted for a different percent of the overall simple size. Thus the difference in ePTB prevalence among the four subgroups identified by the CART model was much larger than that identified by the logistic regression model. Coupling both statistical approaches provides more efficiency for analyzing the overall objective of this study. It also further exemplifies the statistical analysis for similar studies.

Additionally, from a long-term perspective, this pioneering study provides valuable information and direction for our further targeted subgroup enrichment clinical trials aiming at decreasing the prevalence of ePTB among the interactive risk subgroups via supplement pregnant women with DHA.

There are some limitations with this study. Some risk factors contained missing values and/or values of “Not Applicable”, “Unknown,” and “Not Stated,” which added complexity to the proposed analyses. However, data management is unavoidable for any concrete project, and we face the same issue for such a large database regarding birth data for the whole country. The solution taken was from an objective and general perspective, which could deduce the reasonable and acceptable results. Additionally, the risk predictors explored in this paper mainly from mothers’ demographics factors and Maternal pre-pregnancy characteristics, and it does include more highly specific biomarkers. This is due to no such predictors collected in the analysis database. Potentially, this limitation may lead to the relatively low c-index for both models. Further application and reference for these two models should be precautioned.

## Conclusions

This study revealed 14 maternal characteristic variables that can be used reliably to identify risk factor subgroups for ePTB either through a logistic regression model and/or a CART model. Moreover, both models may be used efficiently to identify high risk subgroups for further enrichment clinical trial design.

## Abbreviations

BMI: Body mass index
CART: Classification and regression tree
CDC: Centers for Disease Control and Prevention’s
CI: Confidence intervals
DC: District of Columbia
DHA: Docosahexaenoic acid
ePTB: Early preterm birth
GED: General educational development
NCHS: National Center for Health Statistics
OR: Odds ratios
RR: Risk ratios
SBP: Subgroup birth prevalence
